# A novel method for optimizing epilepsy detection features through multi-domain feature fusion and selection

**DOI:** 10.3389/fncom.2024.1416838

**Published:** 2024-11-19

**Authors:** Guanqing Kong, Shuang Ma, Wei Zhao, Haifeng Wang, Qingxi Fu, Jiuru Wang

**Affiliations:** ^1^Health and Medical Big Data Laboratory, Linyi People's Hospital, Linyi, China; ^2^Shandong Open Laboratory of Data Innovation Application, Linyi People's Hospital Health and Medical Big Data Center, Linyi, China; ^3^School of Information Science and Engineering, Linyi University, Linyi, China

**Keywords:** feature selection, feature fusion, discrete wavelet transform, Welch, particle swarm optimization, Pearson correlation analysis

## Abstract

**Background:**

The methods used to detect epileptic seizures using electroencephalogram (EEG) signals suffer from poor accuracy in feature selection and high redundancy. This problem is addressed through the use of a novel multi-domain feature fusion and selection method (PMPSO).

**Method:**

Discrete Wavelet Transforms (DWT) and Welch are used initially to extract features from different domains, including frequency domain, time-frequency domain, and non-linear domain. The first step in the detection process is to extract important features from different domains, such as frequency domain, time-frequency domain, and non-linear domain, using methods such as Discrete Wavelet Transform (DWT) and Welch. To extract features strongly correlated with epileptic classification detection, an improved particle swarm optimization (PSO) algorithm and Pearson correlation analysis are combined. Finally, Support Vector Machines (SVM), Artificial Neural Networks (ANN), Random Forest (RF) and XGBoost classifiers are used to construct epileptic seizure detection models based on the optimized detection features.

**Result:**

According to experimental results, the proposed method achieves 99.32% accuracy, 99.64% specificity, 99.29% sensitivity, and 99.32% score, respectively.

**Conclusion:**

The detection performance of the three classifiers is compared using 10-fold cross-validation. Surpassing other methods in detection accuracy. Consequently, this optimized method for epilepsy seizure detection enhances the diagnostic accuracy of epilepsy seizures.

## 1 Introduction

In traditional medical epilepsy diagnosis, medical experts rely on their personal experience to visually inspect patients' EEG signals (Tatum, 2014). It is time consuming and analytically demanding to detect epilepsy manually. Medical experts have difficulties interpreting EEG signals due to the non-stationary nature of the signals, which may cause human interpretation errors and disagreements (Oliva and Rosa, 2019). As a result, computer-based methods have gradually replaced traditional medical detection methods, helping medical experts to identify epilepsy-related events in EEG recordings (Li et al., 2020; Vargas et al., 2021; Ramakrishnan and Murugavel, 2019; Türk and Özerdem, 2019).

There are four main challenges involved in implementing automatic epilepsy detection and classification: data preprocessing, feature extraction, feature selection, and classifier design. Recent epilepsy classifications have become increasingly dependent on feature selection, and an efficient feature selection method improves classification accuracy significantly. A low computational efficiency has resulted from manually extracting features from large EEG datasets, even as many developers continue to improve algorithms based on machine learning.

Previously, EEG signal features were extracted manually, using methods such as Wavelet Transform (WT), Short-Time Fourier Transform (STFT), and others to categorize various electrocardiograms and EEG signals. Machine learning, along with the continuous development of artificial intelligence, has spurred the development of automatic epilepsy detection technology. With the introduction of algorithms with decomposition, signal correlation, feature engineering, and other features, detection time and classification accuracy have been shortened and improved (Liu et al., 2021).

In addition to this, the following two unresolved problems exist in the current epilepsy detection research: (1) the time-domain features extracted from the original signals are not sufficient to be used as a feature set for epilepsy detection alone; and (2) the extracted features suffer from the problem of irrelevance and independence from epilepsy detection. A number of studies have proposed methods for solving the above problems, including ICFS, AsyLnCPSO-GA, and GA (Khalid et al., 2014; Wei et al., 2020; Prasetiyowati et al., 2020; Mursalin et al., 2017; Gao et al., 2020; Wang et al., 2022; Omidvar et al., 2021). When using the selected features for classification, the existing methods also have low accuracy and high redundancy.

In order to solve the first problem, this work proposes a method for extracting multidomain features from raw EEG datasets by using discrete wavelet transforms (DWT) and Welch methods to extract time-domain (TD), frequency-domain (FD), time-frequency-domain (TFD), and non-linear features from raw EEG datasets. Due to the large number of extracted features, it leads to overfitting of the classifier and the performance of the classifier is greatly reduced, so it is important to consider that the number of features should be proportional to the cost of training and prediction of the classifier, and the features with high relevance needed by the classifier should be selected (Khalid et al., 2014). For the second question, a feature optimization method (PMPSO) is developed that uses the modified particle swarm algorithm (MPSO) and Pearson's correlation coefficient to select features that are both relevant and independent. Compared with the standard particle swarm optimization algorithm (PSO), MPSO has made improvements in convergence speed and global search capabilities. MPSO introduces a key shrinkage factor ϕ. MPSO realizes efficient search on feature subsets and uses classification accuracy as the fitness function for evaluation. In order to further improve the accuracy of feature selection, MPSO combines the Pearson correlation coefficient to perform a second screening of the initially selected features. By calculating the linear correlation between features and removing redundant features with strong correlation, the final feature set has better independence. This not only reduces the training time of the model, but also improves the overall efficiency of epilepsy detection. By removing irrelevant elements, these techniques can positively affect the performance of constructing classifiers (Wei et al., 2020; Prasetiyowati et al., 2020), using feature selection techniques to compare and improve on different classifiers. The main contributions of this paper can be summarized in the following two points:

The EEG signal is decomposed into sub-bands after fusion using DWT, Welch and STFT methods to extract 35 features in a variety of fields. As significant features in various fields, these features improve classification accuracy greatly.

This paper proposes a new method for feature optimization called PMPSO. This efficient feature optimization method combines the improved Particle Swarm Optimization algorithm (MPSO) with the Pearson correlation coefficient. It aims to eliminate features that are unrelated to epilepsy and those with strong correlations among themselves. The final feature vector is the most representative and optimal.

The remaining sections of this paper are organized as follows: Section 2 introduces some of the developed epilepsy detection methods in related work; Section 3 presents the proposed automatic epilepsy detection method in this paper; Section 4 provides the classification experimental results using this method; finally, Section 5 describes the conclusions drawn from the research and outlines future work.

## 2 Related word

In this section, the work on seizure detection over the past two decades is discussed. This leads to the epilepsy detection method proposed in this paper by analyzing the current state of the work.

### 2.1 Related research on feature selection methods

Feature extraction methods based on raw EEG signals were still mostly manual during this period, with techniques such as WT and STFT widely applied for electrocardiogram and EEG classification. The method proposed by Mursalin et al. (2017) combines an improved Correlation-based Feature Selection (ICFS) with a Random Forest classifier to detect epilepsy. The ICFS was used to select prominent features from the time domain, frequency domain, and entropy-based features for RF classification, with 98.45% accuracy. Based on Approximate Entropy and Recurrence Quantification Analysis combined with Convolutional Neural Networks, Gao et al. (2020) presented an automated method for epilepsy EEG recordings, achieving sensitivity, specificity, and accuracy of 98.84, 99.35, and 99.26%, respectively. According to Wang et al. (2022), an improved PSO and Genetic Algorithm were combined to determine the optimal combination of features for epilepsy seizure detection in a hybrid model. By utilizing a novel Asynchronous Learning Factor Particle Swarm Optimization (AsyLnCPSO) and GA for feature selection, a classification accuracy of 95.35% was achieved. In Omidvar et al. (2021), 55 statistical and entropy-based features were extracted from raw EEG signals using DWT. Using GA for feature selection, they achieved improved accuracy, sensitivity, and specificity of 98.7, 97.5, and 100%, respectively. Haputhanthri et al. (2019, 2020) selected the FS4 feature set based on the correlated feature selection algorithm (CFS), which provided a relatively high level of accuracy when compared to other methods because it contained the mean and standard deviation of the five channels (FT9, P3, Oz, TP9, and FC2). Compared to the PMPSO method proposed in this study, the correlation between features is ignored, and the selected feature set has high redundancy and low independence.

The above-mentioned methods for feature selection have a significant impact on epilepsy detection. These methods still select feature sets that have redundant features and are not optimal. This work proposes a PMPSO method that consists of two feature selection techniques, which can be used to select independent feature sets that have strong correlations and the most representative attributes.

### 2.2 Related research on feature extraction methods

Sriraam and Raghu (2017) extracted 26 features from time domains, frequency domains, information theory, and statistics. On these features, Wilcoxon rank-sum tests were applied based on a 95% significance level, and an optimized SVM classifier reached the highest sensitivity, specificity, and accuracy, respectively, of 94.56, 89.74, and 92.15%. The method proposed by Oliva and Rosa (2021) was based on the binary fusion of three domain features (frequency, time-frequency, and nonlinear), producing a total of 105 features for multiclass classification. The work of Xiong et al. (2022) exploited Pearson correlation coefficients, mutual information, and permutation disalignment index to construct a three-layer network, extracting similar features in each network, and optimizing them based on an improved genetic algorithm. In the CHB-MIT database, the method achieved AC, SP, SE, and F1 of 97.26, 97.55, 96.89, and 97.11%, respectively. In the Siena scalp database, AC, SP, SE, and F1 reached 98.88, 99.13, 98.36, and 98.75%.

In the above-mentioned related works, good results have been achieved in the detection of epilepsy. Although the selected features have strong relevance to epilepsy detection, the correlation between them has not been reflected, and some redundant features remain. Based on the discussion of the above related works, epilepsy seizure detection still has redundant features. To address the existing challenges, this study constructs a comprehensive dual-feature selection method based on multi-task learning. The method extracts crucial features from multiple domains of EEG signals, which directly impacts classification accuracy. Preprocessed EEG signals are decomposed into five subbands (Gamma, Beta, Alpha, Theta, and Delta) using DWT. Specific potential features related to EEG signals' non-linear and dynamic structure are obtained from each subband. The logarithmic sum, mean, mean power, standard deviation, and ratio of absolute mean are extracted. Additionally, Welch's method calculates spectral density estimation features in different frequency bands, extracting 35 features belonging to different domains from the original EEG signals. Then, the feature selection optimization method (PMPSO) combining the MPSO method improved by PSO and the Pearson correlation coefficient is used to select features with high correlation and strong independence. The MPSO method introduces a shrinkage factor ϕ in PSO to overcome its limitation of fast convergence speed but easy to fall into local optimality, so that particles can search collaboratively in the local area, thereby optimizing features with high correlation. The Pearson correlation coefficient is used to perform a secondary screening of these selected features to remove redundant features with strong correlation and further enhance the independence of the features. Finally, SVM, RF, ANN, and XGBoost classifiers classify epilepsy patients, healthy individuals, and epilepsy seizure detection.

## 3 Material and method

**Figure 1:**
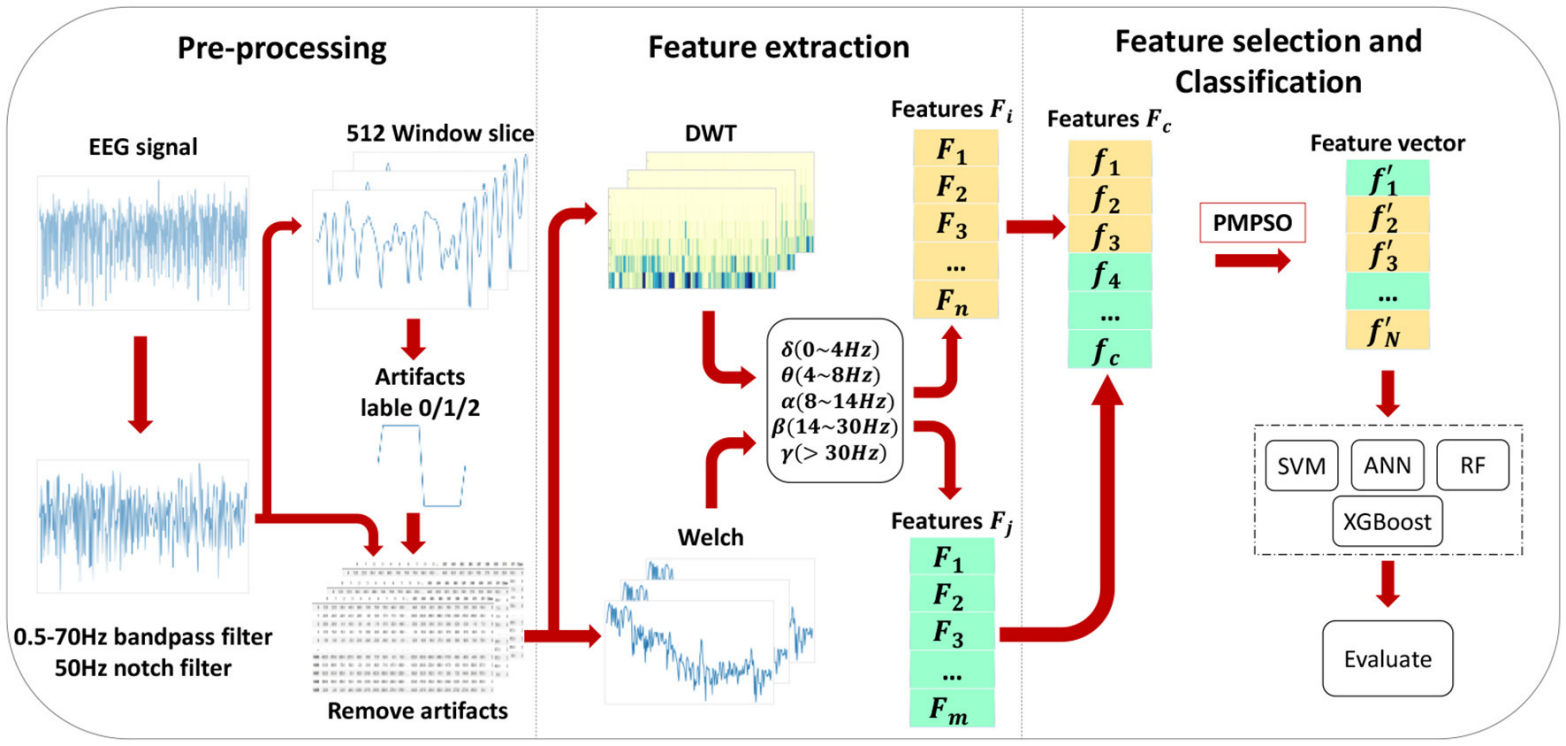
The architectural diagram of the proposed method.

This chapter introduces the proposed epileptic seizure detection system in four parts: raw data preprocessing, feature extraction, feature selection, and classification. Raw data is segmented and filtered in the preprocessing stage. DWT and Welch methods are then applied to EEG segments to extract features from different frequency subbands, resulting in a feature set of 35 features, including important features from various domains, greatly improving classification accuracy. With the PMPSO method, features with strong correlation and independence are selected, forming a representative optimal subset. Finally, the chosen feature subset is serially concatenated to form a feature vector used for training multiple classifiers. Figure 1 illustrates the overall architecture of the proposed method, and the following subsections detail each part of the system.

### 3.1 EEG dataset

In this study, three electroencephalogram (EEG) datasets from different sources were used, namely the epilepsy dataset of the University of Bonn and the CHB-MIT scalp EEG dataset of Boston Children's Hospital. The performance of the PMPSO method was evaluated on these datasets, and the robustness and applicability of the method in different application scenarios were verified.

#### 3.1.1 University of Bonn Epilepsy Dataset

**Figure 2:**
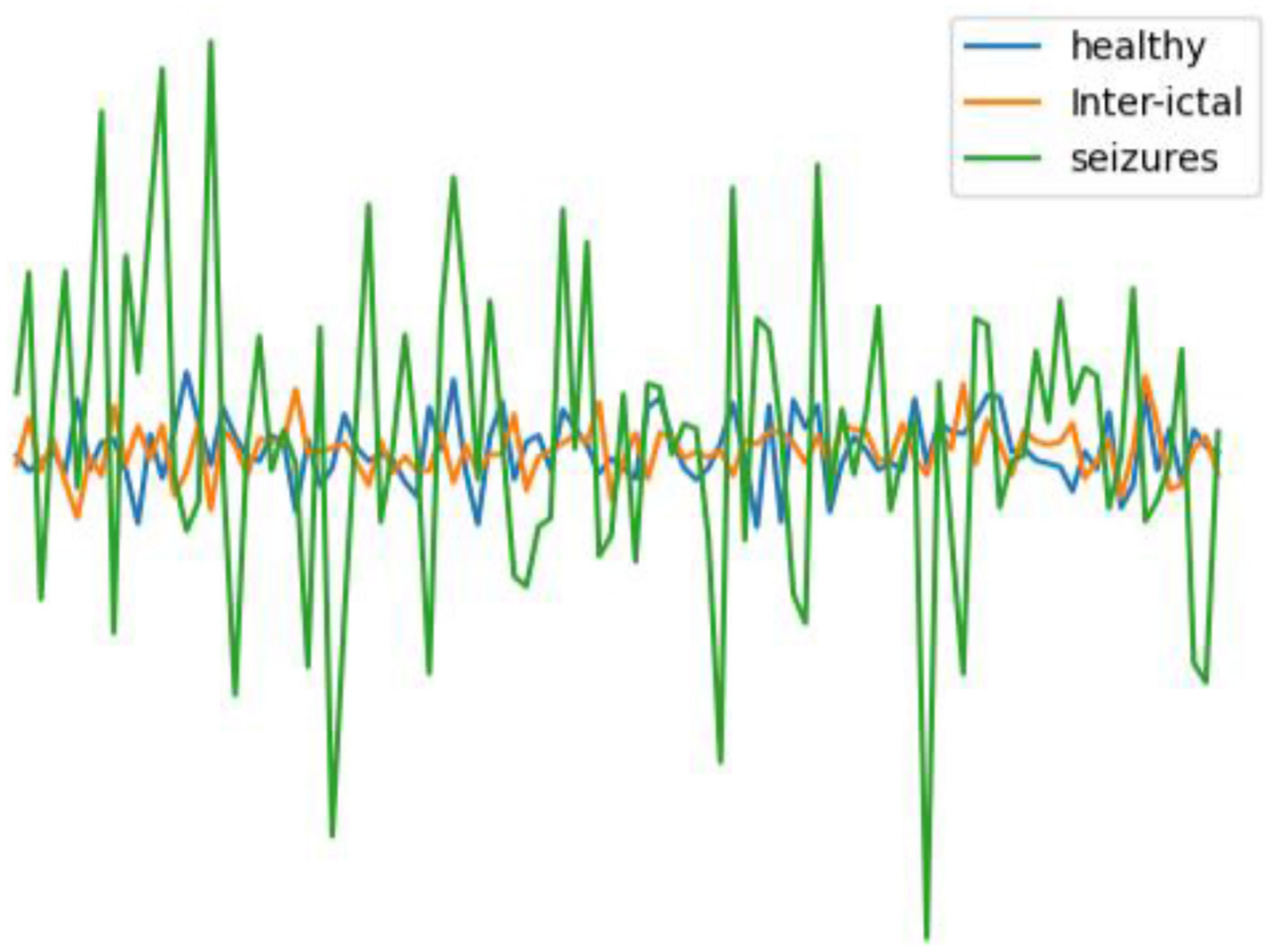
Comparison of EEGs between healthy states, interictal intervals, and ictal periods.

In this work, the epilepsy dataset from the University of Bonn (http://epileptologie-bonn.de/cms/upload/workgroup/lehnertz/eegdata.html) (Andrzejak et al., 2001) was utilized. The dataset comprises EEG data from five epilepsy patients and five healthy individuals, organized into five subsets labeled A to E. Each subset contains 100 single-channel EEG segments with a continuous duration of 23.6 s, containing 4,097 data points. For healthy, interictal, and seizure periods, 200, 200, and 100 data were available, respectively. After undergoing 12-bit analog-to-digital conversion, the data was continuously written to disk at a sampling frequency of 173.61 Hz (Andrzejak et al., 2001). Potential interferences such as muscle artifacts and eye movement artifacts were removed from the data. Figure 2 illustrates EEG contrasts during healthy states, interictal intervals, and ictal periods.

The EEG data collected from subsets N, F, and S are from hippocampal structures and different electrode positions in epilepsy patients' lesions. Interictal EEG subsets N and F are of epileptic patients, and iCtal EEG subset S is of epileptic patients. The Z and O subsets were obtained from five healthy subjects during awake and relaxed states, with Z representing an eyes-open situation and O representing an eyes-closed situation. Table 1 shows sample EEG recordings for five datasets.

**Table 1:** Description of the Bonn University Epilepsy Dataset.

	**Set A**	**Set B**	**Set C**	**Set D**	**Set E**
Participants	Healthy person	Healthy person	Epileptic patients	Epileptic patients	Epileptic patients
State	Open eyes	Close eyes	Interictal	Interictal	Stage of attack
Types	Cephalic cortex	Cephalic cortex	Intracranial	Intracranial	Intracranial
Electrode placements	Scalp	Scalp	Hippocampal structure	Focal area	Focal area

#### 3.1.2 CHB-MIT

This study used the CHB-MIT (https://physionet.org/content/chbmit/1.0.0/), a publicly available scalp EEG database developed by researchers at Boston Children's Hospital and Massachusetts Institute of Technology (Shoeb, 2009). The dataset contains EEG records of 23 pediatric patients with intractable epilepsy, including 5 males with an age range of 3–22 years and 18 females with an age range of 1.5–19 years (Goldberger et al., 2000). The records were labeled by experienced clinicians. The EEG records of each subject contained 9–42 EDF files with a total duration of ~983 h, including 198 epileptic seizure events. All EEG signals were recorded using the international 10-20 bipolar system with a sampling rate of 256 Hz and a resolution of 16 bits. For more details about the CHB-MIT database, see the study by Goldberger et al. (2000), and the relevant case details are shown in Table 2.

**Table 2:** Detailed description of the CHB-MIT database.

**Patient**	**Gender**	**Age**	**Channel number**	**Epilepsy event number**	**Recording duration**
chb01	F	11	22	7	40
chb02	M	11	22	3	35
chb03	F	14	22	7	38
chb04	M	22	22	4	156
chb05	F	7	22	5	39
chb06	F	1.5	22	10	66
chb07	F	14.5	22	3	67
chb08	M	3.5	22	5	20
chb09	F	10	22	4	67
Chb10	M	3	22	7	50
chb11	F	12	22	3	34
chb12	F	2	22	40	23
chb13	F	3	22	12	33
chb14	F	9	22	8	26
chb15	M	16	26	20	40
chb16	F	7	22	10	19
chb17	F	12	22	3	21
chb18	F	18	18	6	35
chb19	F	19	18	3	29
chb20	F	6	22	8	27
chb21	F	13	22	4	32
chb22	F	9	22	3	31
chb23	F	6	22	7	26

Gender: F, female; M, male.

Recording duration: The approximate duration of each case in hours.

### 3.2 Pre-processing

The purpose of this section is to provide a detailed overview of how raw EEG signals are preprocessed. Epileptic seizures and healthy states cannot be distinguished in some studies. Because EEG signals are relatively weak, they are easily disturbed by external factors or human physiological activities. Consequently, it is impossible to distinguish between epileptic seizures and signals from a healthy state, which may adversely affect experimental results (Riccio et al., 2024; Handa et al., 2023; Li et al., 2023; Pandey et al., 2022). In order to ensure data quality and accuracy, a series of preprocessing operations are carried out before extracting features from EEG signals.

Firstly, linear filters are employed to process the EEG signals. A simple fourth-order Butterworth bandpass filter with a range of 0.5–70 Hertz is included. This filter enhances the signal quality by eliminating unwanted frequency components. To suppress interference from power lines, a notch filter at 50 Hertz is also employed.

Besides mitigating artifacts caused by various factors, it is imperative to address the issue of limited data size as well. Continuous EEG data is usually very large, and the available data for the epilepsy data sample is only 500 data instances. Therefore, the long EEG data is segmented into shorter segments using a segmentation strategy. An overlap of 64 data points on the time axis is used with this strategy, using a fixed-size window of 1,024. With this approach, unstable EEG fragments are segmented into shorter, pseudo-stable EEG segments that have similar statistical characteristics. Expanded data for healthy, interictal and seizure periods to 5,700, 5,700, and 2,850.

Finally, the segmented 14,250 EEG fragments are divided into training, validation, and test sets with proportions of 90, 5, and 5%, respectively. The purpose is to facilitate the subsequent training and evaluation of the model. The preprocessing steps provide a reliable foundation for our research by enhancing the quality and applicability of the data.

### 3.3 Feature extraction

**Figure 3:**
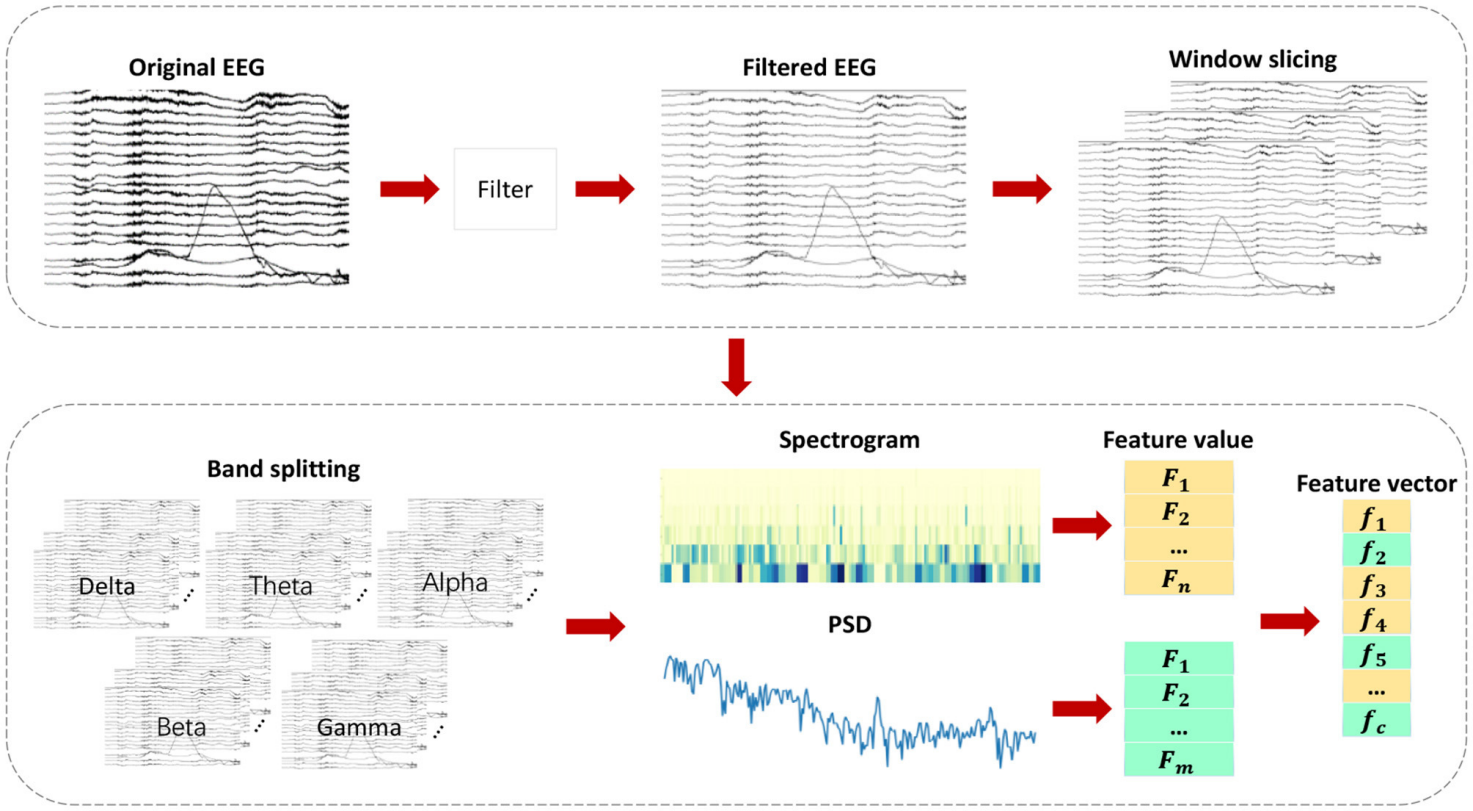
Flowchart of multi-domain feature extraction.

Feature engineering is an essential component in the detection of epileptic seizures. EEG signals during healthy states, ictal periods, and interictal intervals can only be distinguished by extracting significant features from them. As shown in Figure 3, various feature extraction methods are employed in this chapter to extract key features denoted as *f*_1_, *f*_2_, , , *f*_*c*_ from multiple domains. Multi-domain features are extracted by converting the original signal into a format suitable for multi-domain feature extraction, which helps extract key features in each domain. By adding diversity to the feature set, classification accuracy is significantly improved.

The feature extraction process encompasses multiple feature sets from different domains, each having distinct physical and statistical significance. It includes, but is not limited to, time-domain, frequency-domain, time-frequency-domain, and other domain-specific features. As a result of this diverse feature set, our classifier is able to capture various aspects of EEG signals, thereby enabling a comprehensive understanding and differentiation of EEG signals in different states.

#### 3.3.1 Time-frequency domain feature extraction

A time-frequency domain feature extraction is achieved using DWT in this section. Multiple time and frequency scales are used in this method to represent signals in the time-frequency domain through approximation coefficients and detail coefficients. Signal variations can be more accurately described with this approach (Ibrahim et al., 2018). By analyzing the time and frequency information of the signal, extracted time-frequency domain features provide a comprehensive and integrated way to determine the signal's properties. The formula for calculating DWT is shown in equation:


$$DWT\left(i,j\right)=\frac{1}{\sqrt{\left|2^{i}\right|}}\int_{-\infty }^{\infty }x(t)\psi \left(\frac{t-2^{i}k}{2^{j}}\right)\;$$

**Figure 4:**
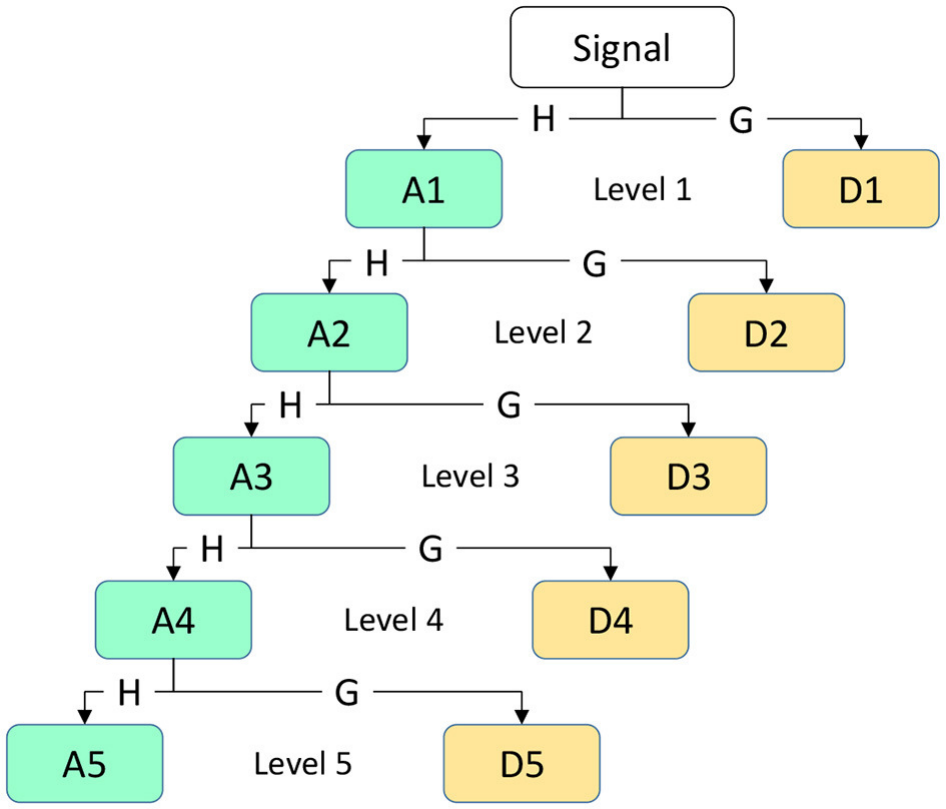
Principle of DWT sub-bands decomposition.

Among them, where *i* represents the frequency band range of the coefficient, *j* represents the position of the wavelet coefficient on the time axis or spatial location, *x*(*t*) represents the original EEG signal, ψ(.) represents the wavelet function (mother wavelet), and *k* is the variable of integration representing the integration across the entire time axis. The primary objective of applying DWT to the EEG signal *x*[*n*] for time-frequency analysis is to extract sub-signals in five different frequency ranges: Delta, theta, alpha, beta, and gamma. In this process, low-pass filters *h*[*n*] and high-pass filters *g*[*n*] are used to generate wavelet coefficients, which are transformed sub-bands. We obtain the approximation coefficient *A*_1_ and the detail coefficient *D*_1_ at the first level. Next, the same procedure is applied to the approximation coefficient *A*_1_ of the first level to obtain the coefficients for the next level. The resulting coefficients *D*_1_,*D*_2_,*D*_3_,*D*_4_,*D*_5_, and *A*_5_ are used to represent EEG sub-bands, as illustrated in Figure 4. This figure depicts a simple diagram of the decomposition of the EEG signal into five coefficients using DWT.

Produce an efficient feature vector by calculating 5 features from each decomposition sub-band. The extracted feature vectors can be reduced in dimensionality by utilizing statistics on discrete wavelet coefficients (Kandaswamy et al., 2004). In this work, the following statistical features were computed using the DWT:

(1) Logarithmic sum of wavelet coefficients (LSWT)

The logarithmic sum of wavelet coefficients refers to taking the logarithm of the absolute values of wavelet coefficients. This aids in capturing information about the signal across different frequency sub-bands. Logarithmic sums can be computed using the following formula:


$$\begin{array}{l} LSWT=\overset{N}{\underset{j=1}{\sum }}ln\left(\left|DWT\left(i,j\right)\right|\right) \end{array}$$

In the aforementioned equation, *DWT*(*i, j*) represents the wavelet coefficient, where *i* indicates the frequency band range of the coefficient, *j* indicates where it occurs in space or on the time axis, and *N* indicates the total energy of the wavelet coefficients in the subband.

(2) The average of the absolute values of coefficients in each subband (MEAN)

The average of the absolute values of coefficients in each subband helps understand the average amplitude of the signal in different frequency ranges. This enables identification of frequency components with significant amplitudes. The formula for calculating the average of the absolute values of coefficients is shown in equation:


$$MEAN=\frac{1}{N}\sum_{j=1}^{N}\left|DWT(i,j)\right|$$

(3) The mean power of wavelet coefficients in each subband (ABS)

The mean power feature refers to the energy distribution of the signal in the frequency domain. It distinguishes the levels of energy within different frequency ranges in the signal. The formula for calculating the mean power of wavelet coefficients is shown in equation:


$$ABS=\frac{1}{N}\sum_{j=1}^{N}{\left(DWT(i,j)\right)}^{2}$$

(4) The standard deviation of coefficients in each subband (STD)

The amplitude distribution and fluctuation characteristics of different types of signals vary from frequency sub-band to frequency sub-band. Standard deviation features help identify and distinguish different types of signals by capturing the characteristics of these amplitude changes. The formula for calculating the standard deviation of coefficients is shown in equation:


$$STD=\sqrt{\frac{1}{N}\sum_{j=1}^{N}{\left(DWT\left(i,j\right)-MEAN\right)}^{2}}$$

(5) The ratio of the absolute average values of adjacent subbands (RAT)

Signal frequency changes can be identified by comparing the absolute average values of adjacent subbands. When the ratio is higher, it indicates that there are more pronounced frequency changes between adjacent subbands, while when it is lower, it indicates relatively small changes in frequency. The calculation formula for the ratio of absolute average values of adjacent subbands is given by equation:


$$RAT=\frac{\sum_{j=1}^{N}\left|DWT\left(i,j\right)\right|}{\sum_{j=1}^{N}\left|DWT\left(i,j+1\right)\right|}$$

#### 3.3.2 Frequency domain feature extraction

Frequency domain features are extracted using the Welch method in this section. An EEG signal's power spectral density (PSD) can be calculated by using this method. Brihadiswaran et al. (2019) summarize different techniques of feature extraction such as statistical feature extraction and entropy based techniques. Compared to these techniques, Welch's method is more suitable for processing EEG signals for epilepsy detection, the extracted features provide information about the energy distribution of the signal in different frequency ranges, which provides a more accurate understanding of the frequency characteristics of the signal (Zhang and Parhi, 2016), with the advantages of fast computation and multi-window selection. Following are the steps to calculate the PSD of EEG signal segments in different frequency bands using Welch's period gram method (Welch, 1967):

First, the EEG brainwave signal *x*(*n*) with a length of *N* is divided into *L* segments, each segment with a length of *M*, where *N* = *ML*. The calculation formula for each segment of EEG signal *x*_*i*_(*n*) is shown in equation:


$$\begin{array}{l} x_{i}\left(n\right)=x\left(n+iM-M\right),0\leq n\leq M,1\leq i\leq L \end{array}$$

Then, use a window function w(n) to mitigate the impact of spectral leakage caused by the edges of time windows on EEG brainwave segments. Calculate the power spectrum of each segment of data using the discrete Fourier transform. The calculation formula is shown in equation:


$$\begin{array}{l} P_{i}\left(w\right)=\frac{1}{U}|\overset{M-1}{\underset{n=0}{\sum }}x_{i}\left(n\right)w\left(n\right)e^{-jwn}{|}^{2},i=1,2,,,L-1 \end{array}$$

Where $U=\frac{1}{M}\overset{M-1}{\underset{n=0}{\sum }}w^{2}\left(n\right)$ is the normalization factor, and *w*(*n*) is the window function.

Finally, the power spectra of all segments are averaged to obtain the power spectrum of the entire signal. This reduces the variance of each power measurement. The calculation formula is shown in:


$$\begin{array}{l} P\left(w\right)=\frac{1}{L}\overset{L}{\underset{i=0}{\sum }}P_{i}\left(w\right) \end{array}$$

This work divided EEG brainwave segments into five frequency bands: delta (0.1–4 Hz), theta (4–8 Hz), alpha (8–12 Hz), beta (12–30 Hz), and gamma (30–70 Hz). The PSD calculation formula for the *i*_*th*_ frequency band is shown in equation:


$$\begin{array}{l} P_{i}=\log\underset{\omega \in bandi}{\sum }P\left(\omega \right) \end{array}$$

As a result of this process, the PSD distribution of EEG signal segments in different frequency ranges can be obtained, which allows a better understanding of the signal's frequency characteristics. The method is widely used in signal processing, spectrum analysis, and frequency domain feature extraction, especially when reducing noise and obtaining smooth spectral estimates are crucial. As shown in Algorithm 1, the pseudocode for extracting PSD features from each sub-band frequency range using the Welch method in this study includes Delta, Theta, Alpha, Beta, and Gamma.

**Algorithm 1:**
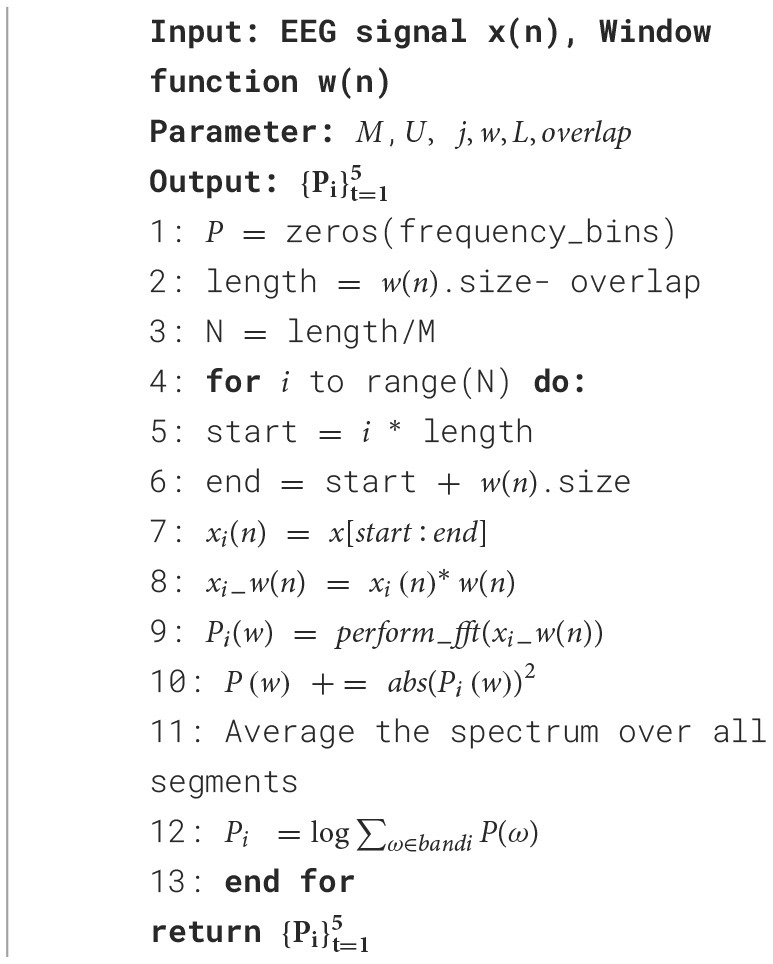
Welch spectrum estimation algorithm.

The calculation of PSD in different frequency ranges follows a methodology similar to the formal description in lines 5–12 of Algorithm 1. Lines 1–3 of Algorithm 1 initialize the parameters. Starting from line 5, the algorithm iteratively applies the window function to each segment of data, preparing for the execution of the Fast Fourier Transform (FFT) to obtain the frequency domain representation of the data. The squared magnitude of the FFT result is calculated in line 10. Finally, the spectral estimates for all segments are accumulated and averaged to obtain the final spectral estimate.

### 3.4 Feature selection

A detailed introduction to the Feature Optimization PMPSO method is provided in this chapter, which is employed to solve the problem of inadequately comprehensive feature selection. A total of 35 features are extracted for each EEG brain signal segment, including 30 time-frequency domain features and five power spectral density features. With the increasing number of features extracted from EEG signals, many irrelevant and redundant pieces of information are present in the time-frequency domain features, resulting in dimensionality catastrophe and a significant impact on the performance of the classifier. An algorithm for selecting features is therefore crucial. In order to reduce feature dimensions and eliminate redundancy, the focus is on selecting those EEG features that most effectively reflect the pre-seizure state. As a result, the classifier's performance and generalization ability are improved. Previous research has only considered one aspect of the correlation, either the correlation between features and seizure occurrence or the correlation among features. Therefore, this section proposes a feature selection method based on MPSO and Pearson correlations. With this method, the MPSO algorithm selects features that are highly correlated with epilepsy detection, thereby minimizing the impacts of irrelevant features on classification results and reducing network overfitting. The Pearson correlation coefficient is employed to calculate the correlation between features. The smaller the correlation between features, the greater the independence of features, leading to a more comprehensive and effective feature set. EEG signals from epileptic patients are better measured with this method (Zhong et al., 2023). In the end, the optimal epilepsy features are obtained after rigorous screening using both methods.

#### 3.4.1 The MPSO algorithm selects features based on their correlation

A MPSO algorithm is presented in this subsection for selecting features with strong correlations. It simulates an individual searching for the best solution in a multi-dimensional space based on the individual best solution (pbest) and the global best solution (gbest), using the PSO algorithm. The particles in PSO represent birds in a flock that move through the search space at a velocity and position. It is the velocity that determines the speed of movement, while the position determines the direction. The individual best is determined independently by each particle in the search space, and this information is shared with all particles. To find the global optimum, particles compare their individual bests with the global best among all particles. Particle speed and position are adjusted based on their individual bests and the current global best. As the iterative process continues, particles collaborate and compete to come up with better solutions.

It is prone to getting stuck in local optima due to the fast convergence of the PSO algorithm. In order to address this issue, a constriction factor ϕ is introduced to limit the range of the factors *c*_1_ and *c*_2_, which control the updating of particle velocity. This helps to reduce the adverse effects that improper learning factor setting may have on the algorithm. The particles will also be able to conduct collaborative search in their immediate vicinity as a result of this. The contraction factor ϕ can be expressed mathematically as follows:


$$\begin{array}{l} \varphi =\frac{2}{\left|2+4c_{1}-\sqrt{2c_{2}^{2}-4c_{1}}\right|} \end{array}$$

Therefore, the updated velocity formula after optimization is given by equation:


$$\begin{array}{l} v_{i}^{t}\left(k+1\right)=wv_{i}^{t}\left(k\right)+\varphi [c_{1}r_{1}(pBest_{i}^{t}\left(k\right)-x_{i}^{t}(k))\\ \;+c_{2}r_{2}(gBest_{i}^{t}\left(k\right)-x_{i}^{t}(k))] \end{array}$$

Here, $v_{i}^{t}\left(k\right)$ is the updated velocity of the *i* particle in the *t* dimension after the *k* iteration. φ is the constriction factor, *c*_1_ and *c*_2_ are the learning factors, *r*_1_ and *r*_2_ are random numbers between 0 and 1 for the k iteration, $pBest_{i}^{t}\left(k\right)$ is the individual best solution of the *i* particle in the *t* dimension after the *k* iteration, $gBest_{i}^{t}\left(k\right)$ is the global best solution in the *t* dimension among all particles after the *k* iteration, $x_{i}^{t}\left(k\right)$ is the current position of the *i* particle in the *t* dimension after the *k* iteration.

In this section, the MPSO algorithm is introduced for finding the optimal feature vector set for epilepsy detection in the feasible space. In Algorithm 2, the optimization process in the feasible space is explained and the algorithm pseudocode is provided. A similar process takes place in the MPSO for computing the optimal solution pbest as described in lines 3–16 of Algorithm 2. In each iteration, fitness values are computed for each particle in steps 3–8, along with individual and global bests. Each particle's position and velocity are then updated in steps 10–17.

**Algorithm 2:**
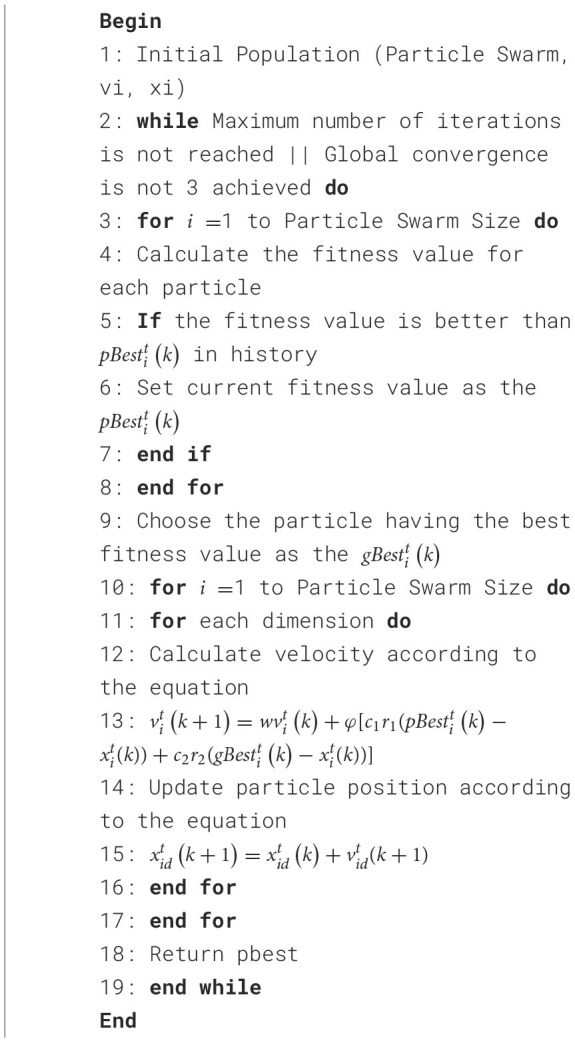
Improved particle swarm optimization (MPSO).

**Figure 5:**
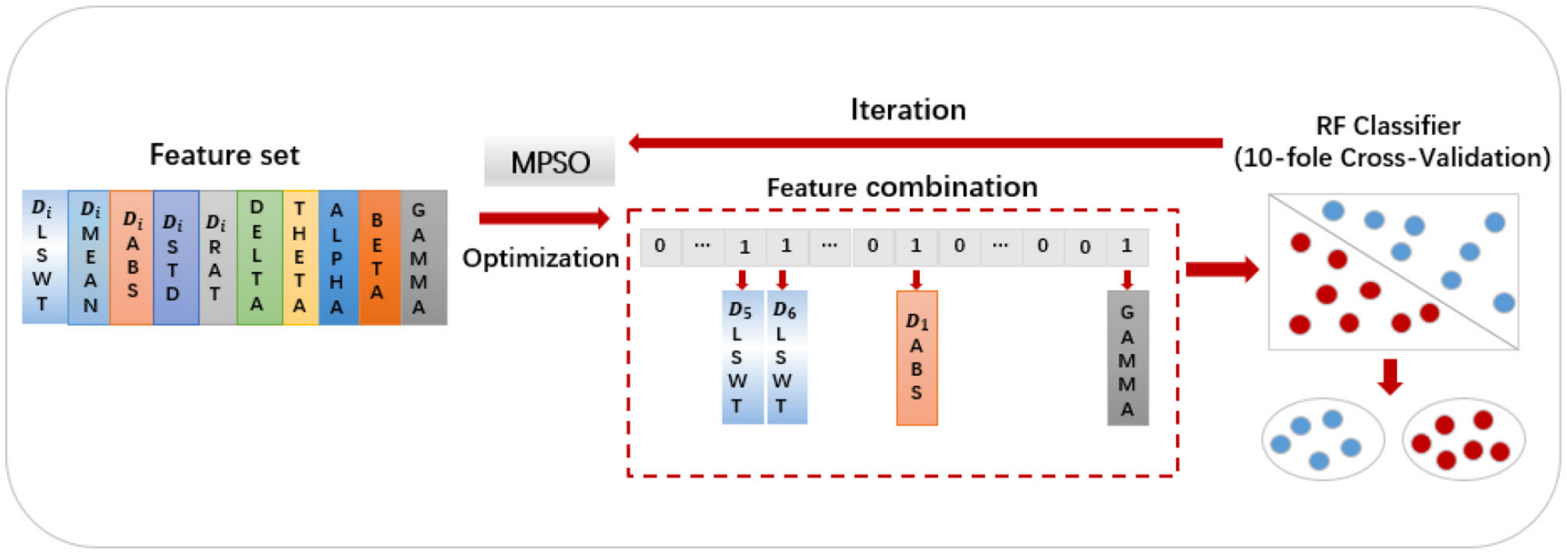
The flowchart of the application of the MPSO algorithm in feature optimization.

MPSO algorithm is applied to feature optimization in the following manner: First, the feature values are sorted in the order of *D*_*i*__*LSWT*, *D*_*i*__*MEAN*, *D*_*i*__*ABS*, *D*_*i*__*STD*, *D*_*i*__*RAT*, *Delta*, *Theta*, *Alpha*, *Beta* and *Gamma*, where *i*ε{1, 2, 3, 4, 5, 6}. A particle swarm optimization algorithm maps particles to binary representations of feature selection statuses. Each extracted feature has two conditions: selected and unselected, represented by 0 and 1, respectively. A binary vector of length 30 composed of 0s and 1s represents each result. As an example, the particle [000011000000100000000000000001] indicates the selection of *D*_5__*LSWT*, *D*_6__*LSWT*, *D*_1__*ABS* and *power*_*gamma*. An algorithm's fitness function is the classifier, and its fitness value is the classification accuracy of each feature combination (Wang et al., 2022). Figure 5 illustrates the detailed process. Each particle's feature values and mapped selection status are initially initialized, with pbest representing the historical best candidate solution for a single particle and gbest representing the population's best candidate solution. These parameters are updated in two scenarios:

(1) The classification performance of the new particle is better than pbest/gbest;
(2) The classification performance of the new particle is the same as pbest/gbest, but the number of features in its corresponding feature subset is smaller, the solution size is smaller.

After reaching the maximum iteration count or finding the global optimum, the iteration ends, and the final feature set selection result is passed on to the next method.

#### 3.4.2 Filtering independent features

The Pearson correlation coefficient is primarily used in this subsection to select features for the second round of analysis. By eliminating features with strong correlations, enhancing feature independence, removing redundant features, and reducing the training time of the model, epilepsy detection becomes more efficient.

The Pearson correlation analysis is widely used to determine the strength and direction of a linear relationship between two variables. Based on the concept of covariance, a correlation coefficient *r* is calculated by dividing two variables' covariances by the product of their standard deviations, resulting in a range of −1 to 1. The formula for calculating the Pearson correlation coefficient *r* is shown in equation:


$$\begin{array}{l} r=\frac{\sum_{i=1}^{n}\left(X_{i}-\bar{X}\right)\left(Y_{i}-\bar{Y}\right)}{\sqrt{\sum_{i=1}^{n}{\left(X_{i}-\bar{X}\right)}^{2}}\sqrt{\sum_{i=1}^{n}{\left(Y_{i}-\bar{Y}\right)}^{2}}} \end{array}$$

The correlation coefficient *r* has a range of values between [−1, +1], and X and Y represent two features. There is a negative correlation when the value is negative, a positive correlation when the value is positive, and no correlation when the value is zero. In general, the closer the correlation coefficient is to 0, the weaker the correlation; the closer it is to −1 or +1, the stronger the correlation.

### 3.5 Applying PMPSO optimized features in the classifier

In this section, we apply the optimal feature vectors extracted using the PMPSO feature optimization method to four different classifiers: ANN, SVM, RF and XGBoost. An ANN consists of an input layer, a hidden layer with 19 neural units, and an output layer with three nodes. For discrete prediction, the softmax output with cross-entropy loss is used, and for real-value prediction, the linear output with square loss is used.

In classification and regression analysis, SVM is a supervised learning method for analyzing data and identifying patterns (Kumar et al., 2017; Vapnik and Cortes, 1995). SVM classification involves separating data points using a hyperplane for input classification (Vapnik and Cortes, 1995). A SVM focuses on support vectors, the data points closest to the decision boundary, which makes it less susceptible to outliers and noise. Complex data can be handled well by SVM because of this property. In order to improve the accuracy of the three-class epilepsy problem, a fifth-order polynomial function is used with adjusted key parameters γ and c. Parameter γ controls the influence range of a single training example on the classification boundary. Parameter c balances correct classification and margin maximization, set to γ = 0.1 and c = 1.

The RF classifier is a machine learning model based on the bagging concept, introduced by Breiman (2001), incorporating additional randomness. A RF model consists of multiple simple decision tree predictors, each of which produces an output based on a set of predictor values. A decision tree is simultaneously constructed by RF by using different bootstrap samples, changing how classification or regression trees are traditionally constructed (Breiman, 2001).

The XGBoost classifier is a tree boosting method. It builds a strong classifier by gradually building multiple weak classifiers (usually decision trees) and combining their predictions. Each step reduces the weight of the previous round of incorrect predictions, allowing the model to gradually learn data points that are difficult to classify. Compared with traditional gradient boosting methods, XGBoost further improves performance through a variety of optimization techniques (Chen and Guestrin, 2016).

## 4 Results and discussion

An analysis of the design and implementation of the proposed epilepsy detection model is presented in this chapter. To work the impact of design on multi-domain feature extraction and PMPSO's performance metrics, experiments were conducted. A statistical analysis of the features extracted using DWT and Welch methods is included in the testing of multi-domain feature extraction. Experiments on the MPSO algorithm for filtering correlations between features and Pearson correlation analysis for determining feature independence are included in PMPSO. Using the random forest classifier, ablation experiments showed that the two proposed methods greatly improved seizure detection efficiency. Four classifiers were used to classify the selected optimal feature vector: ANN, RF, SVM and XGBoost.

### 4.1 Statistical analysis of features

**Figure 6:**
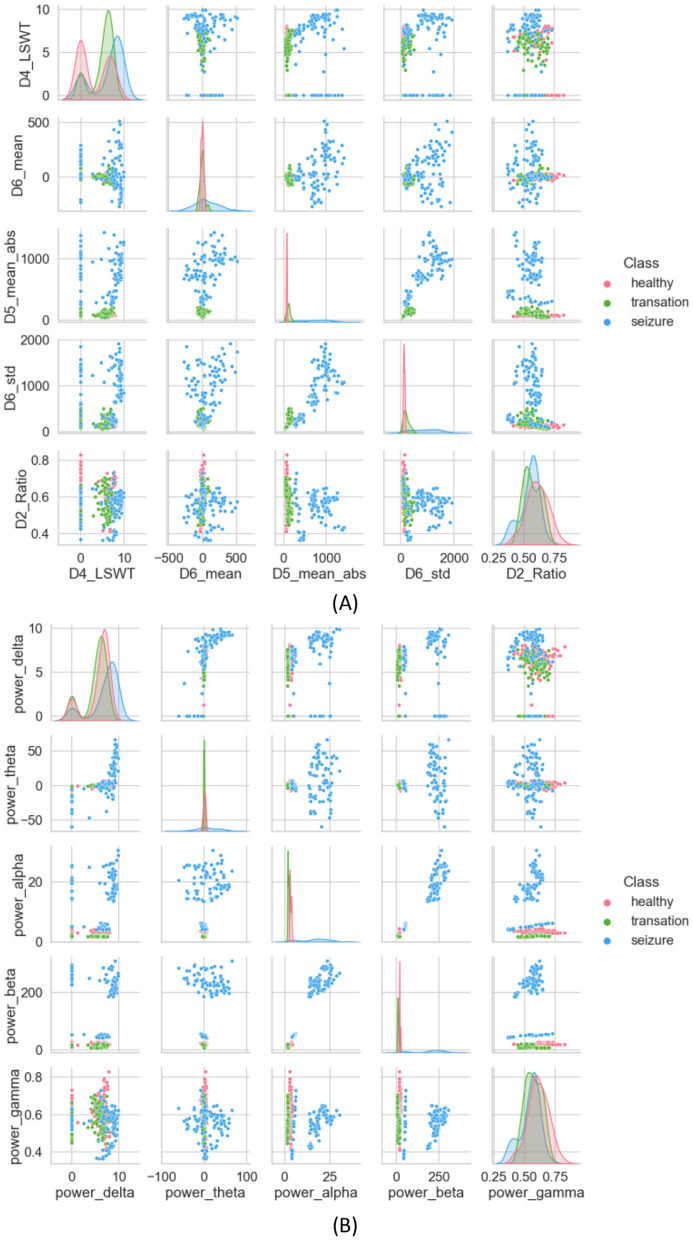
**(A)** Scatter plot of features extracted using the DWT method, **(B)** scatter plot of features extracted using the Welch method.

Under this subsection, 35 features extracted from different domains are analyzed statistically using methods such as DWT and Welch. DWT is initially used to decompose the original EEG signal. A high-pass filter g[n] and a low-pass filter h[n] are used to decompose the original EEG signal into five subbands, represented by coefficients D1, D2, D3, D4, and A4. A number of statistical features are computed for each subband, including LSWT, Mean, ABS, STD, and Ratio. The scatterplot of the proposed features is shown below in Figure 6A, which randomly selects five of the extracted features as a comparison. It is clearer from the scatterplot in Figure 6A that the three categories are separated more clearly. However, a few cluster closer together. The features extracted from seizures are distinctly different from those extracted from healthy and inter-seizure periods.

A PSD is extracted using the Welch method, which estimates frequency-dependent features and aids in understanding static properties that capture both static and dynamic attributes of time-evolving properties, serving as a seizure detection feature (Harpale and Vinayak, 2021). To minimize the loss of edge information during data processing, overlapping time windows are used. For each window, the discrete Fourier transform is applied to calculate the periodicity of the signal. Finally, each period gram is squared before being averaged, reducing the variance of the power spectral density measurements. Figure 6A presents the scatter plot of the calculated average PSD frequency features. In Figure 6B, interictal and ictal periods are clearly distinguished, with little overlap between the two, but the health category is difficult to distinguish.

### 4.2 Application results of MPSO

In this section, features that are closely related to the study are selected using the improved Particle Swarm Optimization algorithm (MPSO). In each iteration of the particle swarm, when updating the particle's position and velocity, the MPSO algorithm introduces a contraction factor ϕ to limit the range of learning factors *c*_1_ and *c*_2_, effectively controlling the change of the velocity vector. Brihadiswaran et al. (2019) summarize some commonly used feature selection techniques such as correlation-based feature selection (CFS), analysis of variance (ANOVA), PCA, and input selection and test training (TWIST). From a multi-domain feature set, the improved MPSO method selects more features relevant for epilepsy detection. By using the contraction factor ϕ, the MPSO method mitigates the effects of improper learning factor settings on the algorithm's performance. The particles are also encouraged to search for solutions collaboratively in the local area. As a result, the MPSO algorithm has a stronger exploratory power and is less likely to fall into local optima than the PSO algorithm.

**Figure 7:**
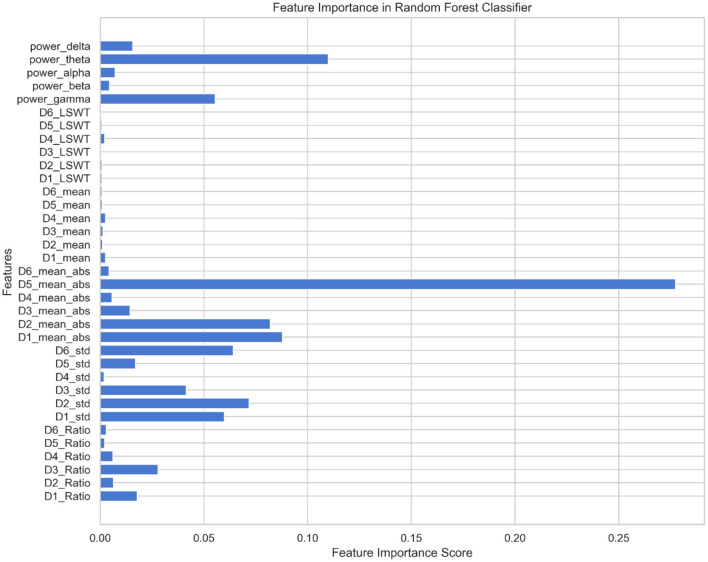
The importance of features in MPSO algorithm.

Using the feature set F as the input for the MPSO algorithm, *F* = {*D*_*i*__*LSWT*, *D*_*i*__*Mean*, *D*_*i*__*ABS*, *D*_*i*__*STD*, *D*_*i*__*Ratio*, *power*_*delta*, *power*_*theta*, *power*_*alpha*, *power*_*beta*, *power*_*gamma*}, where *i*∈{1, 2, 3, 4, 5, 6}, the classification results of the classifier are used as the fitness functions for each feature. The correlation weights of each feature for epilepsy detection are calculated after multiple iterations of optimization, as shown in Figure 7.

As shown in Figure 7, *D*_5__*ABS* and *power*_*theta* have the highest importance for epilepsy classification when using the MPSO method. However, LSWT, Mean, Ratio features computed for each sub-band have a very low percentage of contribution to epilepsy classification and have a significant inhibitory effect. For the next module, the top 10 features with strong correlations are retained based on their correlation weights. In this work, the following 10 features are retained for Pearson correlation analysis: *D*_5__*ABS, power*_*theta, D*_1__*ABS, D*_2__*ABS, D*_2__*STD, D*_6__*STD*, *D*_1__*STD, power*_*gamma, D*_3__*STD, D*_5_<*uscore*>*STD*.

### 4.3 Application results of Pearson correlation analysis

Pearson *r* represents the correlation coefficient between variable *X* and variable *Y* in this section. The value of *r* ranges from −1 to 1, and the expression for *r* is as follows:


$$r=\{\begin{array}{c} 1,perfect\;positive\;correlation\\ -1,\;perfect\;negative\;correlation\\ 0,\;no\;linear\;relationship \end{array}$$

Whenever the correlation coefficient *r* is close to 1, it indicates a strong linear relationship between two variables, *X* and *Y*. In contrast, when the absolute value of the correlation coefficient is close to 0, it indicates that the two variables have no linear relationship, indicating that their variations are not related. A correlation coefficient's sign also indicates the direction of the relationship between variables.

In this work, correlation coefficient *r* was calculated using pairwise combinations of the 10 features mentioned above. Since the number of base features is large, we set the threshold δ at 0.6. A feature was removed from the feature set if its calculated correlation coefficients *r* between feature *X* and feature *Y* exceeded a particular threshold δ. By eliminating that feature from subsequent calculations, computational resources were saved. Table 3 shows the correlation coefficient *r* calculated using Pearson correlation analysis. First, the correlation coefficient r values between *D*_5__*ABS* and *power*_*theta*, *D*_3__*STD*, and *D*_5__*STD* are all above threshold δ. This indicates that this feature's independence is weak, so it is excluded from the feature vector. The features selected later are similar. Finally, a feature vector consisting of *D*_2__*ABS, D*_5__*STD, power*_*gamm, power*_*theta and D*_1__*ABS* was selected as input for the classifier.

**Table 3:** The calculation of the correlation coefficient *r* between features.

	**Features *X***	**Features *Y***	***r***		**Features *X***	**Features *Y***	***r***
1	D_5_ABS	*power*_*theta*	0.712	10	D_1_ABS	D_2_ABS	0.742
2	D_5_ABS	D_1_ABS	0.463	11	D_1_ABS	D_2_STD	0.427
3	D_5_ABS	D_2_ABS	0.546	12	D_1_ABS	D_6_STD	0.413
4	D_5_ABS	D_2_ABS	0.547	13	D_1_ABS	D_1_STD	0.782
5	D_5_ABS	D_6_ABS	0.554	14	D_1_ABS	*power*_*gamm*	0.553
6	D_5_ABS	D_1_ABS	0.488	15	D_2_STD	D_6_STD	0.506
7	D_5_ABS	*power*_*gamma*	0.497	16	D_2_STD	*power*_*gamm*	0.444
8	D_5_ABS	D_3_ABS	0.613	17			
9	D_5_ABS	D_5_ABS	0.795	18			

### 4.4 Performance evaluation

In order to evaluate the effectiveness of the developed algorithm for distinguishing seizure and interictal states, four evaluation metrics will be used to assess its performance. These metrics are SE, SP, AC, and F1. The definitions of these evaluation metrics are as follows:

SE refers to the proportion of actual positive instances that the model correctly identifies.


$$\begin{array}{l} SE=\frac{TP}{TP+FN}\times 100\% \end{array}$$

SP refers to the proportion of actual negative instances that the model correctly identifies among all true negative instances. Specificity describes the model's ability to distinguish negative instances.


$$\begin{array}{l} SP=\frac{TN}{TN+FP}\times 100\% \end{array}$$

AC is the ratio of the number of samples correctly predicted by the model to the total number of samples in all instances. These parameters are defined as follows:


$$\begin{array}{l} AC=\frac{TP+TN}{TP+FN+TN+FP}\times 100\% \end{array}$$

The F1 score is a comprehensive metric for evaluating model performance, commonly used in binary or multiclass classification problems. It combines two key performance metrics: precision and sensitivity. The F1 score is calculated using the following formula:


$$\begin{array}{l} F1=\frac{2^{*}\left(AC^{*}SE\right)}{\left(AC+SE\right)}\times 100\% \end{array}$$

Where *TP* is true positive, *FN* is false negative, *TN* is true negative, and *FP* is false positive. These performance metrics are used to evaluate the performance of the proposed model in this study.

### 4.5 Ablation study

**Figure 8:**
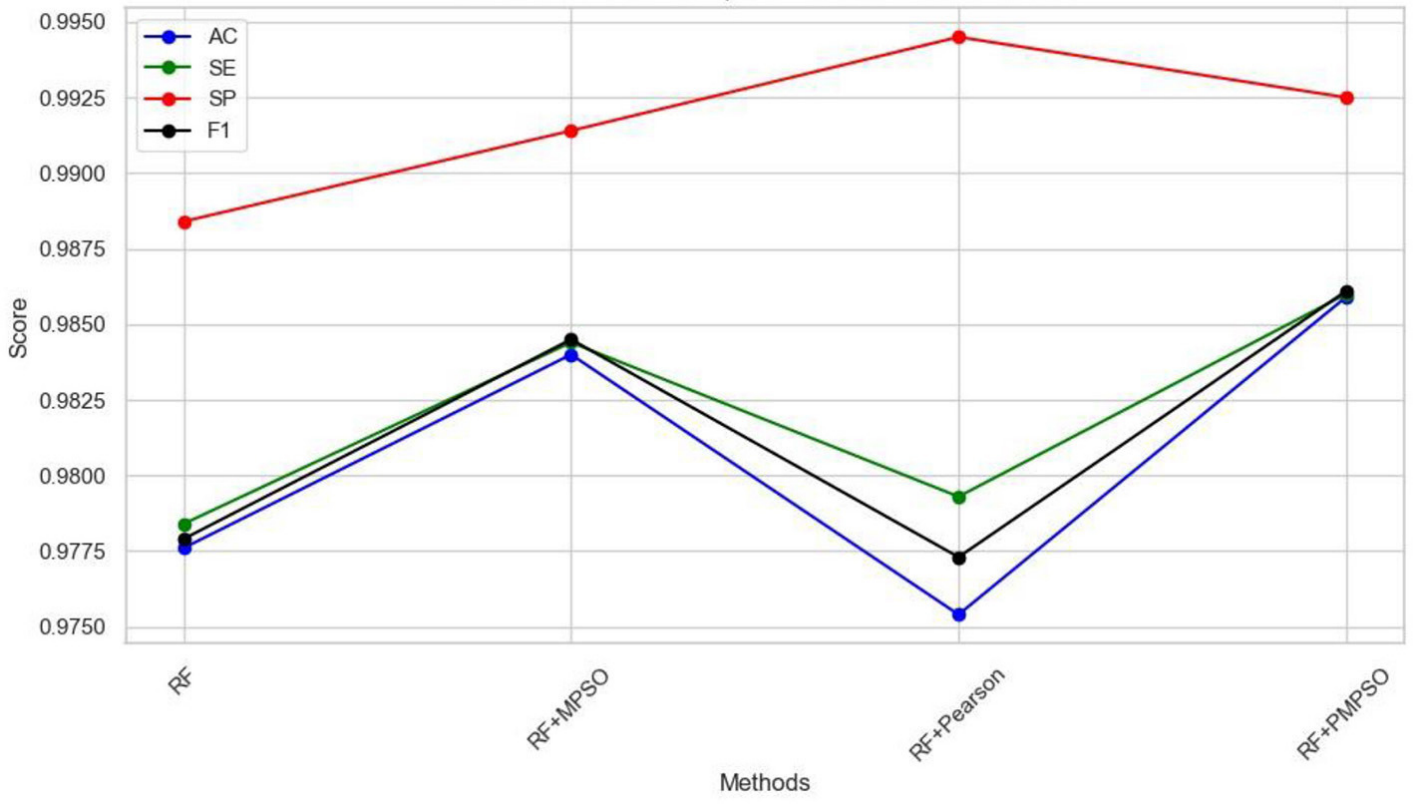
The results of ablative experiments with different methods.

This work aims to use the PMPSO feature optimization method, which consists of MPSO, Pearson, and RF classifiers. In order to analyze the effectiveness of each module in PMPSO, an ablation study was conducted on the Bonn University Epilepsy Dataset, as shown in Figure 8. Specifically, the study derived the following four model variants.

(1) Classification using only the Random Forest classifier.
(2) RF + MPSO: MPSO without Pearson correlation analysis, combined with the Random Forest classifier.
(3) RF + Pearson: Pearson correlation analysis without MPSO, combined with the Random Forest classifier.
(4) RF + PMPSO: Training MPSO and Pearson correlation analysis together.

The following conclusions can be drawn from the ablation study shown in Figure 8 and Table 4. First of all, MPSO can improve classification performance, demonstrating the need to model epileptic seizures in conjunction with feature vectors. In addition, the Pearson correlation analysis improves classification efficiency by removing redundant features from the feature vector by comparing RF and RF + Pearson. In conclusion, PMPSO significantly improves performance in epileptic seizure detection compared to the other three variants in the ablation study. The proposed feature optimization method shows significant performance improvement in epileptic seizure detection.

**Table 4:** The results of the ablation study.

	**AC (%)**	**SE (%)**	**SP (%)**	**F1 (%)**
RF	97.76	97.84	98.84	97.79
RF + MPSO	98.40	98.44	99.14	98.45
RF + Pearson	97.54	97.93	99.45	97.73
RF + PMPSO	98.59	98.60	99.25	98.61

### 4.6 Comparison with baseline methods

In this section, we will compare the PMPSO feature optimization method for epilepsy detection to the baseline PSO method. In the experiment, PMPSO not only introduced the shrinkage factor ϕ to dynamically adjust the search range of the particle swarm, avoiding the problem that the traditional PSO algorithm easily falls into local optimality during the optimization process, but also enhanced the speed and position update process by optimizing the speed and position update process. Global search capabilities of the model. This improvement enables PMPSO to more effectively balance the exploration and exploitation processes, resulting in faster convergence and fewer iterations of feature selection. Compared with the baseline PSO, PMPSO shows stronger robustness and efficiency in feature selection. In addition, PMPSO performs secondary feature screening combined with Pearson correlation coefficient to remove redundant features and improve the independence of features. This dual optimization strategy ensures that feature subsets are more relevant and reduces the possibility of overfitting, thereby significantly improving model performance in classification tasks.

According to Table 5, the improved MPSO method significantly outperforms the baseline PSO method in terms of classification accuracy. With a post-selection feature subset size of 5, PMPSO improves classification accuracy to 98.59%, a 9.09% improvement over the baseline PSO. The precision, recall, and F1 score of PMPSO are also higher than those of baseline PSO, indicating that the feature subset selected by PMPSO can improve the classifier's overall performance. Through its improved feature selection strategy, PMPSO can more efficiently select the features that have a higher contribution to the classification task, improving the model's performance.

**Table 5:** Comparison of classification performance of baseline PSO methods for MPSO and PMPSO feature optimization methods.

**Method**	**Feature subset size**	**AC (%)**	**SP (%)**	**SE (%)**	**F1 (%)**
PSO	11	89.50	87.88	90.12	88.92
MPSO	11	92.34	90.51	93.00	91.57
PMPSO	5	98.59	99.25	98.60	98.61

This Table 6 compares the computational efficiency of PMPSO and baseline PSO methods. Despite the 59.8 s average computation time for PMPSO, which is slightly longer than baseline PSO (47.2 s) mainly due to the dynamic adjustment strategy and the additional shrinkage factor ϕ. In contrast, PMPSO (Dong et al., 2023) requires fewer iterations to complete feature selection than baseline PSO (Sun et al., 2022) suggesting that its optimization process is more efficient and is able to complete feature selection in fewer iterations. Since the sharp stop strategy is implemented during the training process, it is evident from the table that the PMPSO method achieves higher accuracy in fewer training epochs than the baseline PSO.

**Table 6:** Comparison of computational efficiency of baseline PSO methods for MPSO and PMPSO feature optimization methods.

**Method**	**Feature subset size**	**Average calculation time (seconds)**	**Number of feature selection iterations**	**Epoch**
PSO	11	47.2	50	247
MPSO	11	53.6	45	198
PMPSO	5	59.8	40	185

### 4.7 Comparison with state-of-the-art feature selection techniques

In order to verify the effectiveness of the proposed PMPSO method in the feature selection task, this experiment selected the genetic algorithm (GA) and the mutual information-based feature selection method (MIS) for experimental comparison. These methods are widely used in feature selection problems and can effectively reduce redundant features and improve the performance of the classifier. In the epilepsy dataset of the University of Bonn and under the same experimental conditions, PMPSO, GA and MIS were used for feature selection, and the selected features were input into the classifier. In order to ensure the fairness of the experiment, the parameter settings and optimization processes of all methods were kept consistent. The classifier used random forest (RF), and the performance of the model was evaluated by 10-fold cross validation. The experimental evaluation indicators include classification accuracy, the number of features after feature selection, and the running time of the algorithm.

Table 7 shows the performance of the three feature selection methods under different evaluation indicators. From the experimental results, it can be seen that the PMPSO method performs well in classification accuracy, reaching an accuracy of 98.59%, which is significantly better than GA (96.35%) and MIS (95.48%). In addition, the number of features selected by the PMPSO method is relatively small, only 5 features, while GA and MIS select 12 and 15 features, respectively. This shows that PMPSO can effectively remove redundant features while retaining key features, thereby improving the generalization ability of the model. In terms of running time, the average calculation time of PMPSO is 59.8 s, which is better than GA's 85.7 s and slightly higher than MIS's 72.3 s. PMPSO shows significant advantages in accuracy, feature subset size and running time, reflecting its unique innovation and practical application value in feature selection tasks. By introducing an improved particle swarm optimization algorithm, PMPSO demonstrates stronger robustness and higher feature selection efficiency in epilepsy detection tasks, making it an effective supplement and improvement to existing feature selection technology.

**Table 7:** Performance comparison of different feature selection methods.

**Method**	**AC (%)**	**Feature subset size**	**Average calculation time (seconds)**
GA	96.35	12	85.7
MIS	95.48	15	72.3
PMPSO	98.59	5	59.8

### 4.8 Multi-model classification experiments

In this experiment, in order to verify the effectiveness of the PMPSO feature optimization method in epilepsy detection, a multi-model classification experiment was designed, using the Bonn University Epilepsy Dataset and the Boston Children's Hospital CHB-MIT Dataset. The PMPSO method was applied to three common classification models: artificial neural network (ANN), support vector machine (SVM), random forest (RF) and XGBoost. Due to computing resource limitations, the training set of the SVM model was reduced to 2,000 samples on the University of Bonn and CHB-MIT datasets, while the ANN, RF and XGBoost models used the full dataset. A comprehensive experiment was conducted on the three-classification task of epilepsy detection (healthy, interictal, and epileptic seizure).

The experimental results are shown in Table 8. The experimental results on the Bonn University dataset show that the PMPSO method performs very well in different classifiers. The accuracy of ANN, SVM, RF and XGBoost models reached 98.11, 98.25, 98.59 and 99.32% respectively. Among them, the XGBoost model performed best in various evaluation indicators, with a specificity of 99.64%, a sensitivity of 99.29% and an F1 score of 99.32%. In order to enhance the statistical credibility of the results, this paper calculated the 95% confidence interval for each model. The 95% confidence interval of the classification accuracy of the XGBoost model is (98.61, 99.75), while the 95% confidence intervals of the accuracy of the RF, ANN and SVM models are (97.80, 99.38), (97.20, 99.02), and (97.30, 99.18), respectively, which further proves the stability of the PMPSO method.

**Table 8:** Classification results of the PMPSO feature optimization method.

**Classifier**	**AC (%)**	**SP (%)**	**SE (%)**	**F1 (%)**	**95% CI for AC(%)**
ANN	98.11	98.54	98.61	98.36	(97.20, 99.02)
SVM	98.25	98.93	98.91	98.57	(97.30, 99.18)
RF	98.59	99.25	98.60	98.61	(97.80, 99.38)
XGBoost	99.32	99.64	99.29	99.32	(98.61, 99.75)

Experiments on the CHB-MIT dataset further validated the robustness of PMPSO. The original EEG signal of each patient was used as the input signal of the algorithm. The classification results for each patient were calculated and the average evaluation parameters were reported, as shown in Table 9. On the EEG data of 23 pediatric patients with intractable epilepsy, the accuracy on the RF classifier was 98.42%, the specificity was 98.83%, the sensitivity was 98.61%, and the F1 score was 98.57%. The confidence interval of the accuracy of the RF classifier on the CHB-MIT dataset is (97.90, 98.95), which shows the consistency of the performance of this method on different patient data.

**Table 9:** Classification results of PMPSO feature optimization method on CHB-MIT dataset.

**Patient**	**AC (%)**	**SP (%)**	**SE (%)**	**F1 (%)**	**95% CI for AC(%)**
chb01	97.92	98.27	97.89	98.07	(96.80, 99.04)
chb02	98.33	98.67	98.41	98.37	(97.40, 99.26)
chb03	98.74	98.99	98.69	98.71	(97.82, 99.66)
chb04	98.09	98.31	98.02	98.13	(97.05, 99.12)
chb05	98.63	98.89	98.53	98.58	(97.70, 99.56)
chb06	99.03	99.21	98.92	99.01	(98.10, 99.94)
chb07	98.19	98.43	98.07	98.12	(97.14, 99.21)
chb08	97.94	98.29	98.01	97.96	(96.92, 98.97)
chb09	98.68	98.91	98.57	98.61	(97.81, 99.55)
chb10	98.23	98.59	98.18	98.21	(97.30, 99.18)
chb11	98.51	98.76	98.44	98.49	(97.64, 99.45)
chb12	98.42	98.83	98.61	98.57	(97.70, 99.34)
chb13	98.86	99.07	98.79	98.81	(97.99, 99.66)
chb14	97.97	98.24	98.08	98.02	(96.82, 99.12)
chb15	98.69	98.96	98.64	98.66	(97.80, 99.56)
chb16	97.93	98.18	97.86	97.94	(96.81, 99.05)
chb17	99.12	99.29	99.04	99.07	(98.22, 99.96)
chb18	98.71	98.93	98.64	98.67	(97.85, 99.61)
chb19	97.89	98.12	97.79	97.88	(96.76, 99.04)
chb20	98.91	99.14	98.82	98.86	(97.95, 99.72)
chb21	98.43	98.72	98.31	98.38	(97.51, 99.32)
chb22	98.04	98.32	97.96	98.09	(96.94, 99.13)
chb23	98.57	98.84	98.52	98.56	(97.71, 99.51)
Average	98.42	98.83	98.61	98.57	(97.90, 98.95)

**Figure 9:**
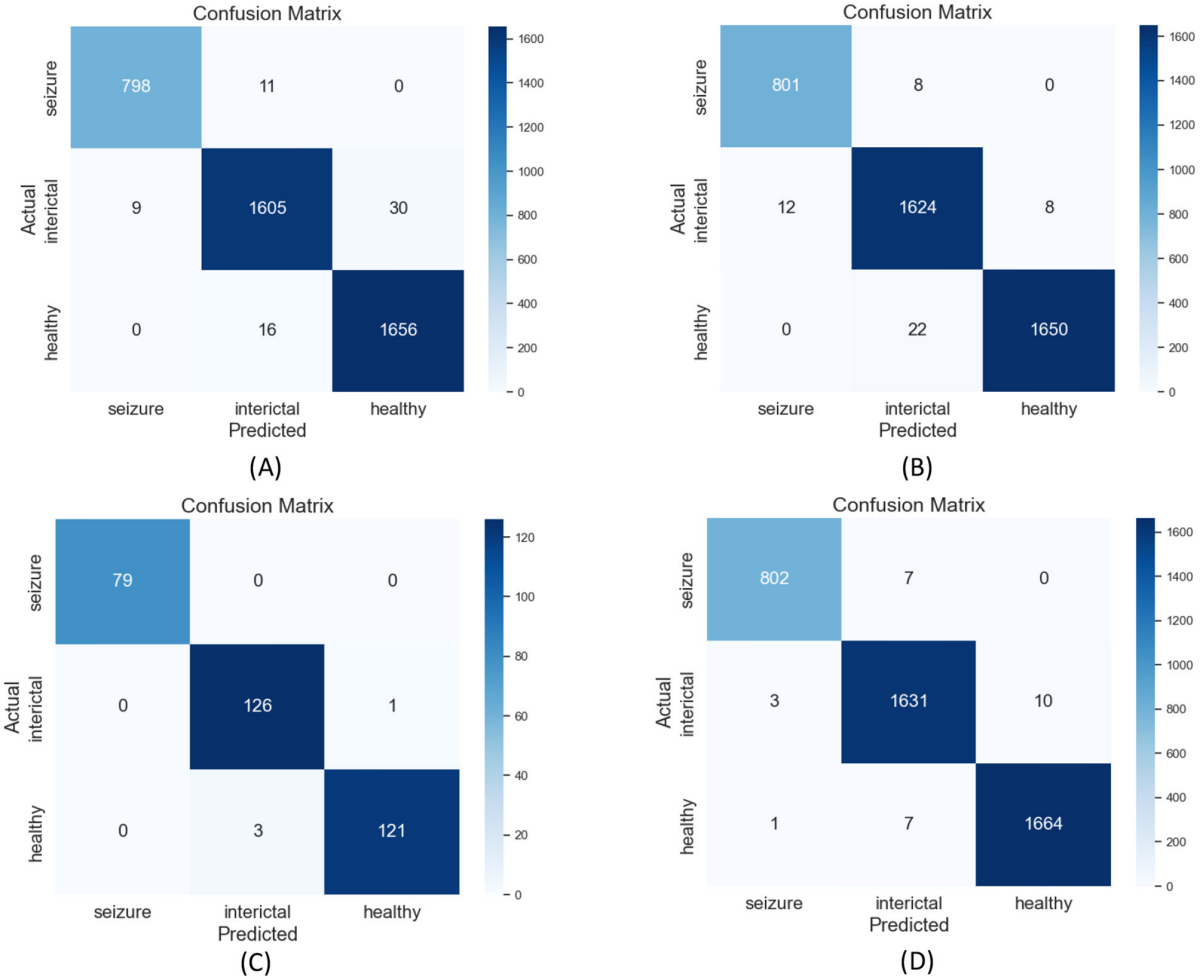
Confusion matrix plots for **(A)** RF, **(B)** ANN, **(C)** SVM, and **(D)** XGBoost classification using the Bonn epilepsy dataset.

**Figure 10:**
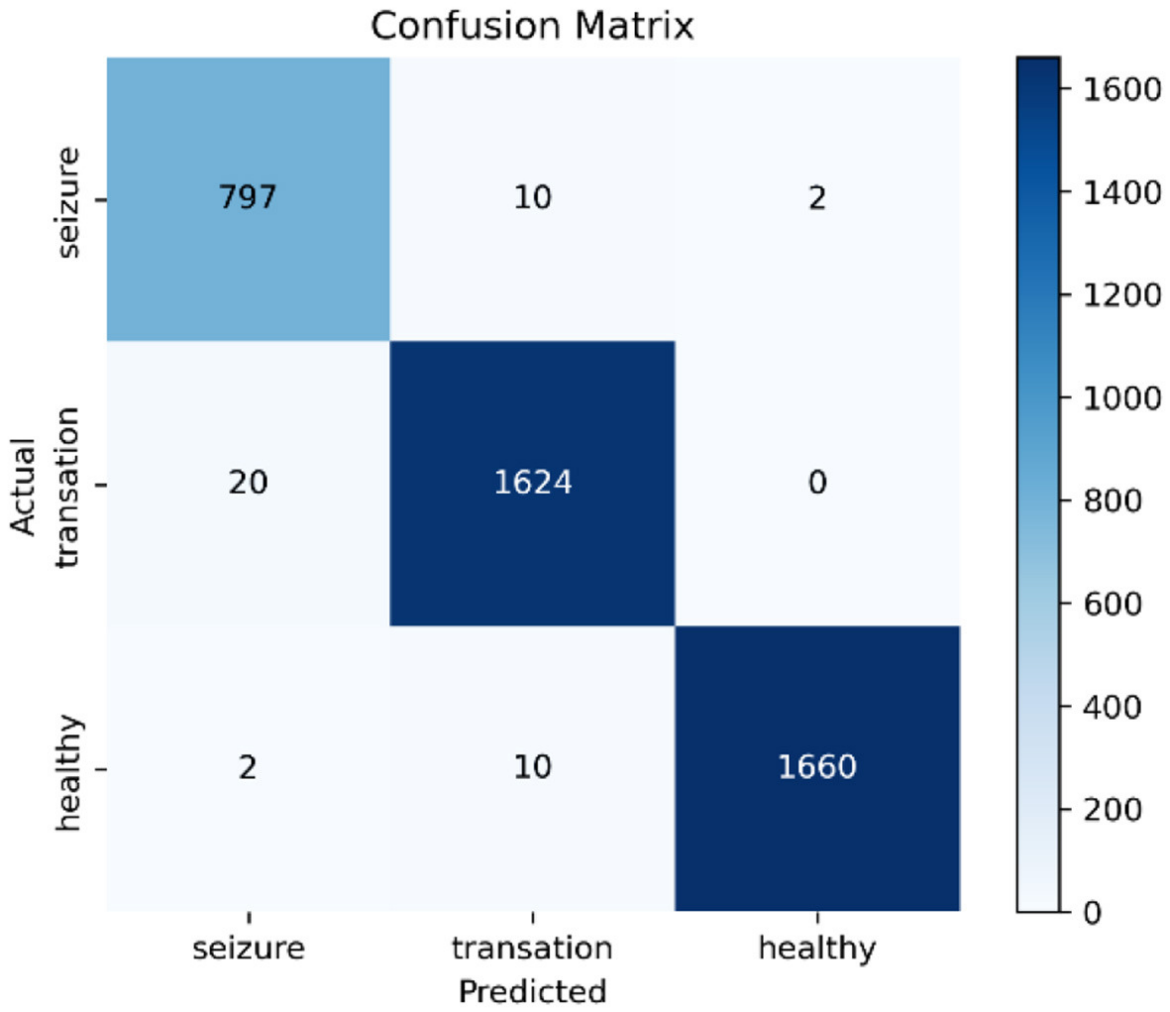
Confusion matrix of the RF classifier using the CHB-MIT dataset.

Additionally, confusion matrix plots were generated using evaluation metrics, illustrating the accuracy, sensitivity, and specificity of the four classifiers. In Figures 9, 10, you can clearly see the specific classification of health, epileptic seizures, and interictal periods. The current work on epilepsy detection is compared to recent research in Tables 10, 11. Using different feature extraction and selection methods, we significantly improved classification accuracy and efficiency under the same dataset conditions. Compared to other algorithms, the proposed algorithm shows substantial improvements in performance, as shown in Tables 10, 11.

**Table 10:** Performance comparison of different methods on the Bonn database.

**Method**	**AC (%)**	**SP (%)**	**SE (%)**	**F1 (%)**
Wang et al. (2022)	95.35	–	–	–
Xiong et al. (2022)	97.26	97.55	96.89	97.07
Sairamya et al. (2021)	95.74	95.73	95.74	95.74
Bhanot et al. (2020)	93.4	93	93	93.2
Mouleeshuwarapprabu and Kasthuri (2023)	97.29	97.41	97.77	97.53
Majzoub et al. (2023)	98.23	97.50	97.50	–
Cai et al. (2024)	98.00	98.18	98.18	–
Song et al. (2024)	98.52	98.30	98.88	98.57
PMPSO	99.32	99.64	99.29	99.32

**Table 11:** Comparison of state-of-the-art epileptic seizure detection methods evaluated using the CHB-MIT dataset.

**Method**	**AC (%)**	**SP (%)**	**SE (%)**	**F1 (%)**
Harpale and Bairagi (2021)	96.48	95.34	96.52	95.93
Zarei and Asl (2021)	97.09	97.26	96.81	97.03
Li et al. (2021)	97.47	97.50	97.34	97.42
Sun et al. (2022)	98.14	98.64	96.79	97.71
Cimr et al. (2024)	98.30	97.32	97.90	97.61
PMPSO	98.42	98.83	98.61	98.57

## 5 Conclusion

By focusing on feature extraction and feature selection, this paper explores an effective approach to epilepsy seizure detection using EEG data. First, 35 measurement features were extracted using methods such as DWT and Welch. Thereafter, a novel feature optimization method, Particle Swarm Optimization with Modified Shrinkage Factor (PMPSO), was developed to select features that are more relevant to epilepsy detection, reducing feature redundancy and enhancing detection efficiency and accuracy. A four-classifier approach was used to evaluate selected feature subsets: ANN, SVM, RF, and XGBoost. Finally, the results were assessed through 10-fold cross-validation. The experiments demonstrated better performance than previous works that overlooked feature selection and relied on deep learning methods. The introduced model benefited from the application of computational techniques in feature selection, enhancing the signal processing aspect of machine learning methods. The results validate the proposed approach by significantly reducing the computational workload while achieving comparable results through a substantial reduction in the number of features extracted from EEG segments.

For future work, the following plans are outlined: (1) Proposing feature selection techniques more suitable for epilepsy seizure detection; (2) Further reducing the number of features while maintaining or improving classification accuracy, aiming to use the minimum number of features for optimal classification efficiency; (3) Evaluating the model on epilepsy signal databases with more channels.

## Data Availability

The original contributions presented in the study are included in the article/supplementary material, further inquiries can be directed to the corresponding author.
